# Performance of Density Functional Approximations in Calculations of Electronic Two-Photon Transition Strengths of Fluorescent Dyes

**DOI:** 10.1021/acs.jpca.5c01509

**Published:** 2025-05-24

**Authors:** Marta Chołuj

**Affiliations:** Department of Physical and Quantum Chemistry, Faculty of Chemistry, Wroclaw University of Science and Technology, Wybrzeze Wyspiańskiego 27, Wrocław 50-370, Poland

## Abstract

The performance of B3LYP, PBE0, and CAM-B3LYP functionals in the prediction of the two-photon transition strengths (for low-energy transitions) of 18 dipolar donor–acceptor systems containing a difluoroborate moiety was evaluated against results obtained using the resolution-of-identity implementation of the coupled-cluster CC2 model. The generalized few-state model approach, in which the two-photon transition strength is expressed in terms of electronic structure parameters, i.e., excitation energies, dipole moments, and transition dipole moments, was applied to gain deeper insight into the behavior of selected exchange-correlation functionals. The obtained results show that all three functionals provide two-photon transition strengths that differ significantly from the reference strengths, especially in the case of molecules exhibiting the highest 2PA strength.

## Graphical abstract

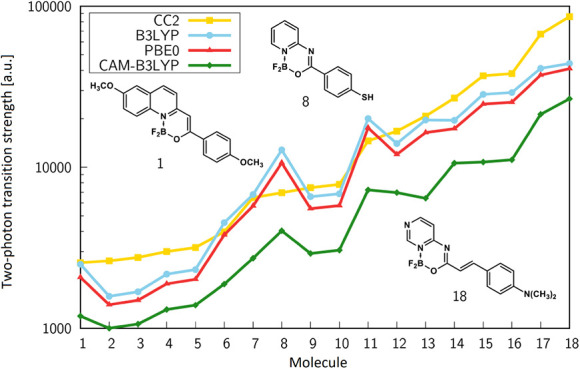

## Introduction

Nonlinear optics is a branch of optics that studies interactions between high-intensity light, as generated by lasers, and matter.^1^ The origins of nonlinear optics date back to 1931, when Maria Göppert-Mayer described the first nonlinear optical phenomenon, i.e., two-photon absorption (2PA), in her doctoral thesis.^2^ However, it was the invention of the laser by Theodore Maiman in 1960 that initiated the intensive development of research in this area and made it possible to observe nonlinear effects.^3^

For the strong electric field of the light wave with intensity greater than 10^8^ V/m (nowadays achieved by short-pulse lasers), the microscopic description of optical response assumes that the relation between the induced dipole moment (μ_ind_) and the intensity of the electric field (*F*) becomes nonlinear, and the *i*th component of the induced dipole moment (μ_*i*_(*F*)) is represented by the following equation:

1 $$\mu_{i}\left(F\right)=\mu_{i}\left(0\right)+\underset{j}{\sum }\alpha_{ij}F_{j}+\frac{1}{2!}\underset{j,k}{\sum }\beta_{ijk}F_{j}F_{k}+\frac{1}{3!}\underset{j,k,l}{\sum }\gamma_{ijkl}F_{j}F_{k}F_{l}+\cdot \cdot \cdot$$

where μ_*i*_(0) and *F*_*i*_ denote *i*th component of the permanent dipole moment and *i*th component of the electric field, respectively, whereas *i*, *j*, *k*, *l* ∈ {*x*, *y*, *z*}. The above equation defines fundamental optical properties, i.e., polarizability (α) as well as first-, second-, and higher-order hyperpolarizabilities (β, γ, ...). Hyperpolarizabilities are linked to nonlinear optical effects. For instance, the first hyperpolarizability (β) is associated with second harmonic generation, the Pockels effect, sum frequency generation, and optical rectification, whereas the second hyperpolarizability (γ) is related to two-photon absorption, third harmonic generation, and the optical Kerr effect.^1^

One of the best-known and most frequently studied phenomenon of nonlinear optics is the two-photon absorption, in which two photons are simultaneously absorbed, leading to the excitation of a chemical object to a higher energy state.^1,4,5^ Years of research resulted in numerous technological applications that utilize this phenomenon, among which one can mention bioimaging,^6–10^ photodynamic therapy,^11–13^ microfabrication,^14^ three-dimensional optical data storage,^15,16^ and optical power limiting.^17^ Technological advances initiated the search for molecular systems with a large two-photon optical response and ways to enhance this response. The main challenge here is to understand the relationship between the structure of the chemical object and its optical properties. Examples of systems with high 2PA potential are organic, organometallic, and dendrimeric molecules of dipolar, quadrupolar, or octupolar structure^4,18^ as well as metal–organic frameworks, perovskites, and materials with reduced dimensionality (particularly 2D materials).^5,19–21^ At this stage, it should be mentioned that there are also other factors known to influence 2PA response of molecular objects, i.e., solvent polarity,^22^ bond length alternation,^23,24^ spatial confinement,^25–27^ and long-range charge-transfer processes.^28^ Despite the vast amount of work devoted to two-photon absorption, research in this area continues, as there are still many aspects that require further exploration. It should be emphasized that theoretical chemistry plays a very important role in this field. Quantum-chemical calculations allow one to predict optical properties and perform screenings of various sets of molecules in search of these with high 2PA responses. Moreover, theoretical chemistry offers tools for a thorough study of the structure–property relationship, which helps to elucidate experimental results and supports the design of materials with desired properties. In the theoretical studies of the two-photon absorption, the standard approach is to use response theory combined with coupled-cluster (CC) wave function. In particular, the approximate coupled-cluster singles and doubles (CC2) model is often employed for 2PA analysis.^29–34^ Unfortunately, due to the high computational cost, this approach cannot always be applied to very large molecular systems that are of interest for actual 2PA applications. In such a situation, density functional theory (DFT) is the method of choice. However, there are many exchange-correlation functionals that can be used in 2PA calculations, and therefore, finding the right one, that reproduces the results given by higher levels of theory (CC) or experimental measurements, is a huge challenge. For this reason, benchmark studies that show the behavior of many different functionals in the description of 2PA response of various molecular systems are extremely valuable source of information.^29,31,35^ Note that among many currently available exchange-correlation functionals, the range-separated CAM-B3LYP is very often used for analyzing 2PA of large molecular systems, because in many cases it correctly maps the trends of changes of the two-photon optical response in a group of compounds, compared to the results obtained from calculations performed with CC2 method; however, it usually provides values that are underestimated in comparison to CC2 reference.^31,35^ In the previous work done by the author of the present study, the two-photon absorption strengths of 48 π-conjugated molecules containing electron-donating/-accepting moieties were evaluated using BLYP, PBE, B3LYP, PBE0, CAM-B3LYP, LC-BLYP, and optimally tuned LC-BLYP functionals and compared afterward to CC2 reference.^31^ The obtained results showed that global hybrids (PBE0 and B3LYP) are the most successful in reproducing the absolute values of 2PA strengths. In turn, CAM-B3LYP and optimally tuned LC-BLYP significantly underestimate the 2PA strengths of the analyzed set of compounds. Beerepoot et al. found that the range-separated LC-BLYP and CAM-B3LYP are the only two out of six selected exchange-correlation functionals that predict the correct trends of changes of 2PA strengths for six four-coordinate organoboron N,C chelates in comparison with experimental and CC2 results, although they underestimate the absolute values of 2PA strengths by a factor of 2–6, depending on the molecule considered.^29^ Note that the CC2 values obtained in this work are in good agreement with the experimental data available for three of the studied molecules, which supports applying this method as a reference for evaluating the performance of exchange-correlation functionals in predicting 2PA strengths. On the contrary, Grabarek and Andruniów in their benchmark study on fluorescent protein chromophores, evaluated five different exchange-correlation functionals (BLYP, B3LYP, B1LYP, BH&HLYP, and CAM-B3LYP) against the CC2 reference and found that CAM-B3LYP gives the most accurate 2PA strengths.^30^ In very recent work done by Naim et al., an extensive analysis of the performance of various wave function methods and five commonly used exchange-correlation functionals in the studies of 2PA strengths with respect to the quadratic response implementation of the third-order coupled cluster method including iterative triples (Q-CC3) reference was conducted for a set of 25 small organic molecules.^36^ The obtained results showed that CCSD methods are the most accurate, whereas the quadratic response implementation of the CC2 method (Q-CC2) and second-order algebraic diagrammatic construction in its intermediate state representation (I-ADC2) exhibit much worse performance. Among the evaluated exchange-correlation functionals, BH&HLYP, and CAM-B3LYP were shown to provide the most accurate results, whereas B3LYP was found to be the worst-performing functional. Nevertheless, quantum chemists still put much effort into designing new exchange-correlation functionals and into finding the best one for particular applications. Therefore, the benchmark studies of two-photon absorption hold a strong spot in modern theoretical chemistry. The present work fits this trend, as its goal is to evaluate the performance of exchange-correlation functionals in the description of the two-photon transition strength (δ^2PA^) of selected fluorescent dyes presenting a difluoroborate (BF_2_) group. Note that such compounds are used as probes for two-photon microscopy and ways for tailoring their two-photon properties for this and many other applications were thoroughly analyzed.^37,38^ The present work can serve as a valuable source of information for future theoretical studies, as it provides some interesting observations about the behavior of the exchange-correlation functionals in the description of the two-photon absorption strengths in comparison to the CC2 reference. However, it should be noted that the calculations performed in this benchmark were done in a vacuum, and the obtained results may not be directly transferable to experimental conditions (e.g., molecular systems in solutions).

## Methods

For the purpose of this study, we selected 18 dye molecules containing a difluoroborate moiety (presented in Figure 1). The geometries of these compounds were optimized in the gas phase at the CAM-B3LYP/6-31G* level of theory using the GAUSSIAN 16 program.^39^ A pruned (99,590) integration grid was used, and tight convergence criteria were set on energy gradients during the geometry optimization procedure. The calculations of the spectroscopic properties were carried out at the optimized geometries using three exchange-correlation functionals, i.e., B3LYP,^40–42^ PBE0,^43^ and CAM-B3LYP^44^ in connection with the aug-cc-pVDZ basis set^45^ and a pruned (99,590) integration grid. For that purpose, the GAMESS US program^46^ was employed. B3LYP, PBE0, and CAM-B3LYP functionals are one of the most widely used in the studies of the optical properties of various molecular compounds. Especially, CAM-B3LYP gained popularity in theoretical simulations of the two-photon absorption spectra of extended π-conjugated systems because, alongside other range-separated functionals, it improves the accuracy of excitation energies to Rydberg and charge-transfer states.^47–51^ To evaluate the performance of the selected functionals in the description of the two-photon transition strength, the calculations were performed using the resolution-of-identity implementation of the coupled-cluster CC2 model with the aid of the TURBOMOLE program.^52,53^ The aug-cc-pVDZ basis set^45^ and the corresponding recommended auxiliary basis set^54^ were used for these computations. Note that the studied molecules present donor–acceptor topology and exhibit intramolecular charge transfer upon excitation to the lowest-energy state.

**Figure 1:**
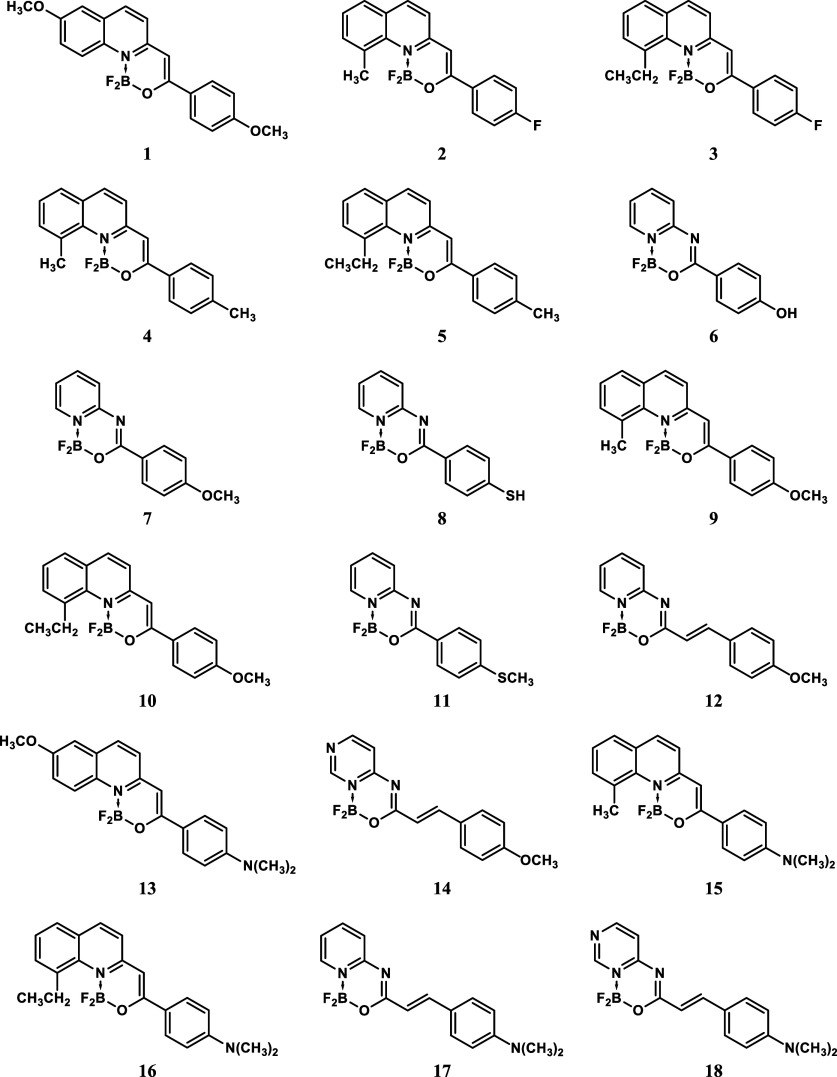
Structures of the studied molecules.

The two-photon transition strengths of all 18 molecules were also analyzed in terms of a generalized few-state model (GFSM) at the CC2 and DFT levels. At this stage, it is worth emphasizing that this approach allows one to express the two-photon transition strength in terms of electronic structure parameters, i.e., excitation energies, dipole moments, and transition dipole moments. Therefore, by using GFSM, one can get insight into the nature of the 2PA process. Within the GFSM approximation, the expression for 2PA strength for non-Hermitian theories, where left and right transition dipole moments are different, is as follows:^29^

2 $$\begin{array}{ll} \delta_{0JKL}^{GFSM} & =\underset{K}{\sum }\underset{L}{\sum }\frac{2}{15\Delta E_{K}\Delta E_{L}}\left(\alpha +\beta \right),\\ \alpha & =\left|\mu^{JK}\right|\left|\mu^{K0}\right|\left|\mu^{0L}\right|\left|\mu^{LJ}\right|(\cos\theta_{JK}^{K0}\cos\theta_{0L}^{LJ}+\cos\theta_{JK}^{0L}\cos\theta_{K0}^{LJ}+\cos\theta_{JK}^{LJ}\cos\theta_{K0}^{0L})\\ \beta & =\left|\mu^{JL}\right|\left|\mu^{L0}\right|\left|\mu^{0K}\right|\left|\mu^{KJ}\right|(\cos\theta_{JL}^{L0}\cos\theta_{0K}^{KJ}+\cos\theta_{JL}^{0K}\cos\theta_{L0}^{KJ}+\cos\theta_{JL}^{KJ}\cos\theta_{L0}^{0K}) \end{array}$$

In the above equation, the superscripts *L*0 and 0*L* denote the right and left transition dipole moment, respectively, $\Delta E_{K}=\frac{1}{2}\omega_{J}-\omega_{K}$ and $\theta_{PQ}^{RS}$ represents the angle between the transition dipole moment vectors μ^*PQ*^ and μ^*RS*^. The corresponding expression for theories with Hermitian structure is given by

3 $$\delta_{0JKL}^{GFSM}=\underset{K}{\sum }\underset{L}{\sum }\frac{4}{15\Delta E_{K}\Delta E_{L}}\left|\mu^{0K}\right|\left|\mu^{KJ}\right|\left|\mu^{0L}\right|\left|\mu^{LJ}\right|\cdot \left(\cos\theta_{JK}^{0K}\cos\theta_{0L}^{LJ}+\cos\theta_{JK}^{0L}\cos\theta_{0K}^{LJ}+\cos\theta_{JK}^{LJ}\cos\theta_{0K}^{0L}\right)$$

One can choose any number of intermediate states *K* and *L* in the generalized few-state model expressions in eqs 2 and 3. In the present study, we will employ a two-state model (2SM) that includes only the ground state 0 and the final excited state 1 and four three-state models with additional states 2, 3, 4, or 5 (SM (2), 3SM (3), 3SM (4), 3SM (5), respectively). Note that for the CC2 method, left and right transition dipole moments are different. Moreover, in some cases, they can even differ in sign, which leads to negative values of oscillator strengths. To ensure the correctness of the results obtained using the generalized few-state models at the CC2 level, the sign differences in the left and right transition dipole moments were checked.

## Results and Discussion

Figure 2 presents the two-photon transition strengths computed with response theory using the CC2 reference method and selected exchange-correlation functionals for molecules **1**–**18**, whereas in Figure 3 a comparison of δ^2PA^ values from CC2 and DFT calculations, by means of the ratios between δ^2PA^(DFT) and δ^2PA^(CC2), is demonstrated. As can be seen from Figure 2, the studied compounds were ordered according to increasing values of δ^2PA^ obtained at the CC2/aug-cc-pVDZ level of theory. The results predicted by the B3LYP, PBE0, and CAM-B3LYP functionals do not follow the growing trend on passing from molecule **1** to **18**. Indeed, the two-photon transition strengths, computed using all three functionals, demonstrate nonmonotonic behavior with respect to the CC2 reference. However, the qualitative trends predicted by B3LYP, PBE0, and CAM-B3LYP are quite similar. Among the considered functionals, the largest values of δ^2PA^ across the whole set of molecules are provided by B3LYP, whereas the smallest ones by CAM-B3LYP. Based on the data presented in Figures 2 and 3 one can notice that CAM-B3LYP underestimates δ^2PA^ of all studied molecules and gives results that differ the most from the reference ones, i.e., from δ^2PA^ obtained at the CC2 level. In fact, the two-photon transition strengths computed using the remaining functionals are also smaller than the corresponding CC2 values for almost all compounds, except **6**, **7**, **8**, and **11**. The best agreement with the CC2 method is observed for B3LYP; however, in many cases, these results are far from satisfactory. In general, there are significant differences between the two-photon transition strengths predicted by the CC2 method and DFT, especially for molecules exhibiting the highest 2PA strengths, i.e., **14**–**18**. For instance, the differences between δ^2PA^(CC2) and δ^2PA^(DFT) for compounds **17** and **18** exceed 20,000 au (see Figure 2 and ).

**Figure 2:**
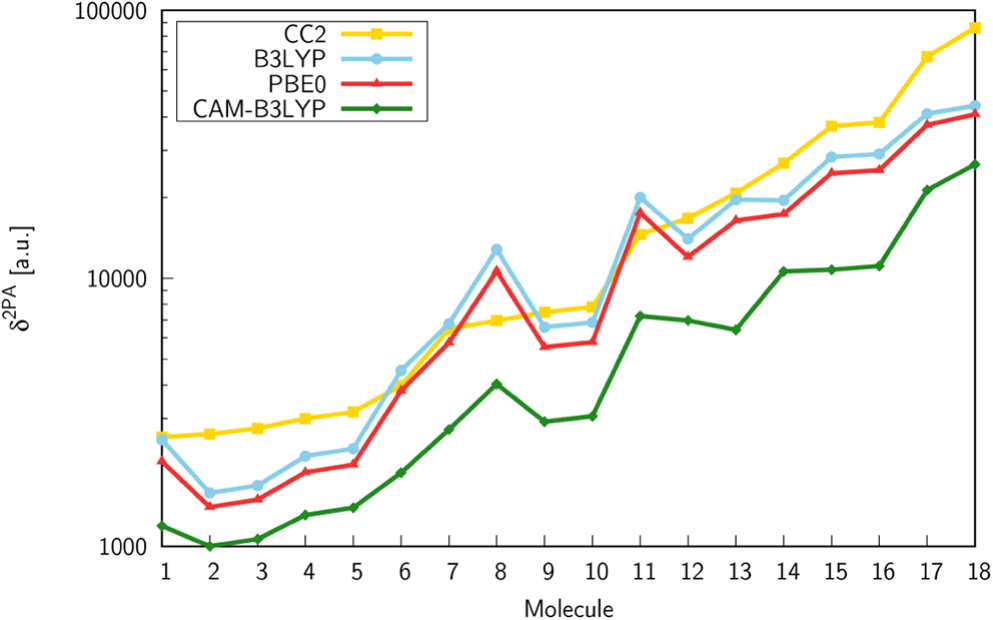
Two-photon transition strengths (δ^2PA^) computed with response theory using the aug-cc-pVDZ basis set.

**Figure 3:**
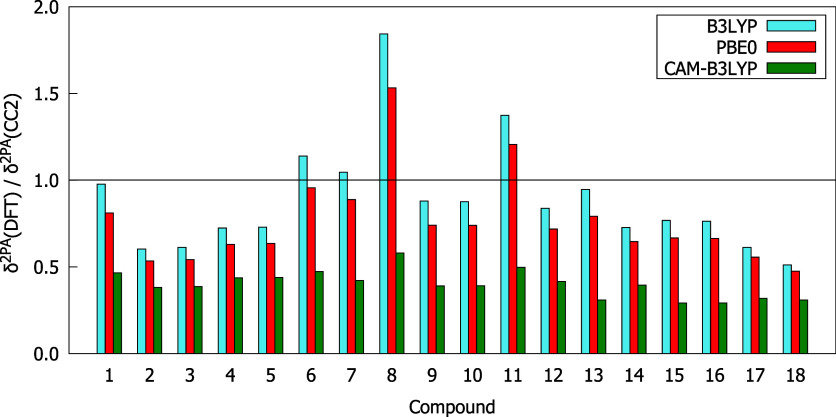
δ^2PA^(DFT)/δ^2PA^(CC2) values computed with response theory using the aug-cc-pVDZ basis set.

To gain insight into the origins of the behavior of exchange-correlation functionals in the description of δ^2PA^ of the studied molecules, we employed a generalized few-state model. First, we compared δ^2PA^ from a two-state model (2SM) that includes only the ground state 0 and the final excited state 1 and four three-state models with additional states 2, 3, 4, or 5 (SM (2), 3SM (3), 3SM (4), 3SM (5), respectively) with response theory (RSP) results. The ratios between δ^2PA^ values obtained using few-state models (FSM) and response theory at the CC2 and DFT levels are presented in . The collected data clearly show that for both CC2 and DFT the two-state model provides results closest on average to those predicted by response theory. The three-state models significantly overestimate the δ^2PA^ of some compounds. For this reason, the two-state model was selected for further investigations.

As can be seen from , which demonstrates the results based on a two-state model in comparison to response theory, the values of δ^2PA^(2SM) are in most cases higher than δ^2PA^(RSP). The only two exceptions were identified for the CC2 method, and these are molecules **9** and **10**. It was also observed that for the vast majority of studied compounds, the 2SM results come closest to the values of δ^2PA^ predicted by response theory in the case of CC2. In fact, a two-state model provides two-photon absorption strengths similar to those obtained from response theory for both CC2 and CAM-B3LYP (0.9 < δ^2PA^(2SM)/δ^2PA^(RSP) < 1.5). In turn, the values of δ^2PA^(2SM) predicted by B3LYP and PBE0 are significantly higher than the corresponding RSP outcomes, with a ratio between δ^2PA^(2SM) and δ^2PA^(RSP) exceeding 2 for some molecules.

Keeping in mind that the two-state model results for B3LYP and PBE0 are not satisfactory, we will now proceed with the analysis of terms contributing to the two-photon transition strength within the two-state approximation, i.e., δ_0001_, δ_0011_, and δ_0111_ (hereafter referred to as δ_00_, δ_01_, and δ_11_, respectively). collects the percentage contributions of these terms to δ^2PA^(2SM). Note that because δ_01_ is equal to δ_10_, their sum was presented in as one contribution. For the vast majority of studied molecules, i.e., **4**–**18**, the values of δ_11_, provided by the CC2 method as well as by B3LYP and PBE0 functionals, prevail over the remaining two terms. On the other hand, the results obtained using CAM-B3LYP show that, depending on the compound in question, δ_01_ + δ_10_ or δ_11_ dominates. Therefore, a proper description of these two terms is important for a correct determination of δ^2PA^(2SM) and, if the two-state model provides reliable results, also for δ^2PA^(RSP). In most cases, δ_00_ has the smallest contribution to δ^2PA^(2SM).

A comparison of the δ_00_, δ_01_, and δ_11_ values obtained using the CC2 method and the exchange-correlation functionals is demonstrated in Figures 4–6. It is apparent that all three functionals come quite close to the δ_00_ values predicted by CC2 with a ratio between δ_00_(DFT) and δ_00_(CC2) ranging from 0.6 to 1.5. Only slightly worse behavior of DFT was observed in the description of δ_01_ for most molecules. However, it should be noted that the δ_01_ of compound **6** is significantly underestimated by PBE0 and CAM-B3LYP, whereas the values of δ_01_ provided by B3LYP and PBE0 for compound **8** are more than two times larger than the corresponding CC2 results. The CC2 method predicts a negative sign of δ_01_ for almost all molecules, apart from **6**, **8**, and **11**. The analyzed functionals give the sign of δ_01_ consistent with the reference data with only two exceptions, i.e., PBE0 and CAM-B3LYP for compound **6**. The values of δ_11_ based on DFT differ the most on average from the CC2 outcomes in comparison to the remaining contributions to δ^2PA^(2SM). In most cases, B3LYP and PBE0 overestimate δ_11_ (except molecules **14**, **17**, and **18**), whereas CAM-B3LYP underestimates it (except molecules **2** and **3**).

**Figure 4:**
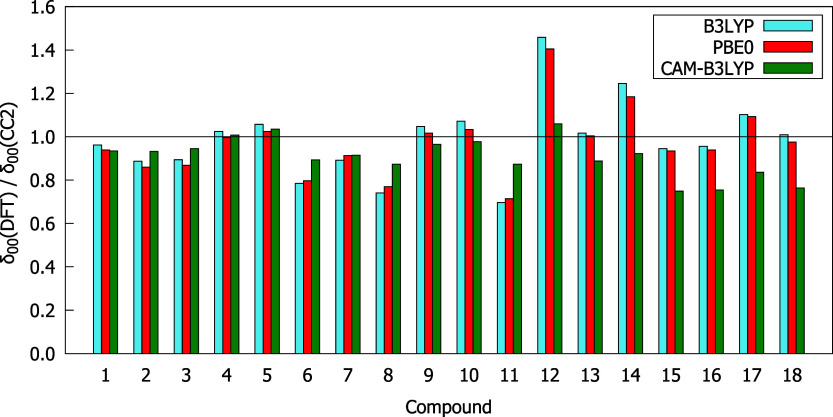
δ_00_(DFT)/δ_00_(CC2) values obtained using the aug-cc-pVDZ basis set.

**5:**
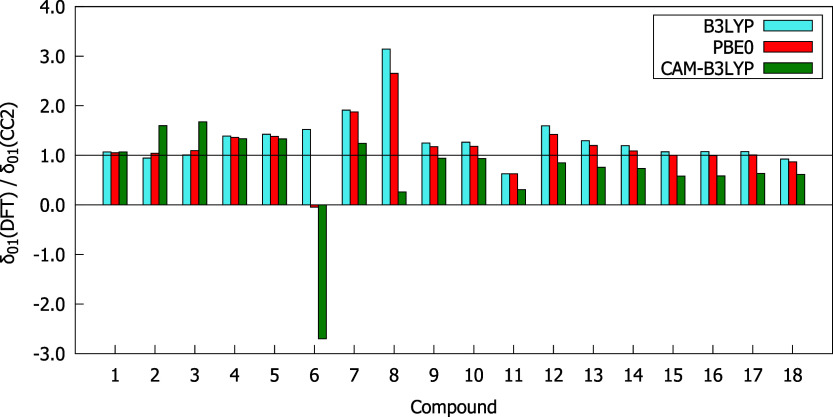
δ_01_(DFT)/δ_01_(CC2) values obtained using the aug-cc-pVDZ basis set.

**Figure 6:**
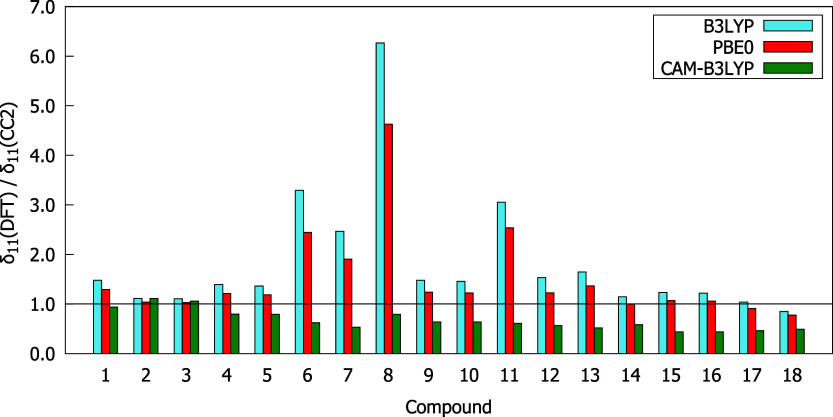
δ_11_(DFT)/δ_11_(CC2) values obtained using the aug-cc-pVDZ basis set.

Particular attention should be paid to molecules exhibiting the highest δ^2PA^, i.e., **13**–**18**. It is clear from Figures 4–6 that δ_00_, δ_01_, and δ_11_ of all these molecules are underestimated by CAM-B3LYP. In turn, the remaining functionals do not show such regularity in the description of these three terms because, depending on the molecule in question, the results of calculations performed with B3LYP and PBE0 are smaller or greater than those obtained from the CC2 method. Although B3LYP and PBE0 give results much closer to the reference ones, the systematic behavior of CAM-B3LYP in predicting δ_00_, δ_01_, and δ_11_ favors this functional in the studies of δ^2PA^(2SM) for molecules with high 2PA strength, because it allows one to avoid error cancellation. This also applies to the calculations of the response-theory value of δ^2PA^, as we have previously shown that in the case of CAM-B3LYP a two-state model is a reliable approximation.

We will now take a closer look at molecules with high 2PA strength, i.e., **14**–**18**, by analyzing their spectroscopic parameters: ground-state dipole moment (|μ_00_|), transition dipole moment (due to the fact that right and left transition dipole moments differ for non-Hermitian theories, we will discuss their multiplication: $\left|\mu_{01\cdot 10}\right|=\sqrt{\mu_{x}^{01}\mu_{x}^{10}+\mu_{y}^{01}\mu_{y}^{10}+\mu_{z}^{01}\mu_{z}^{10}}$ ), excited-state dipole moment (|μ_11_|) and excitation energy (Δ*E*_01_). Figure 7 demonstrates that the values of |μ_00_| are overestimated by B3LYP, PBE0, and CAM-B3LYP. On the other hand, all three functionals underestimate |μ_01·10_| of compounds **14**–**18**. Nonetheless, in both cases, the results obtained using DFT are in good agreement with the CC2 data, and the values predicted by CAM-B3LYP come closest to the reference ones. The ratio between |μ_11_|(DFT) and |μ_11_|(CC2) falls on average further from 1 than in the case of |μ_00_| and |μ_01·10_|. For almost all molecules except **18**, |μ_11_| is overestimated by B3LYP and PBE0. In turn, CAM-B3LYP systematically underestimates |μ_11_| and predicts values that differ the most from the CC2 results. Among the considered functionals, PBE0 works best in the description of |μ_11_|. Similarly to |μ_00_| and |μ_01·10_|, the exchange-correlation functionals give values of Δ*E*_01_ very close to the values of the CC2 method. B3LYP and PBE0 underestimate the excitation energies, whereas CAM-B3LYP overestimates them. At this stage, it is important to note that the absolute values of δ_01_ + δ_10_ and δ_11_ obtained using CAM-B3LYP for compounds **14**–**18** are very similar and that these two terms contribute the most to δ^2PA^(2SM) (see ). According to eq 2, |μ_00_|, |μ_11_|, and Δ*E*_01_ directly contribute to δ_01_ and δ_11_. Therefore, we can pinpoint the underestimated values of the excited-state dipole moment and overestimated excitation energies as potentially the main sources of the underestimation of δ_01_ and δ_11_ and hence also δ^2PA^(RSP) by CAM-B3LYP.

**Figure 7:**
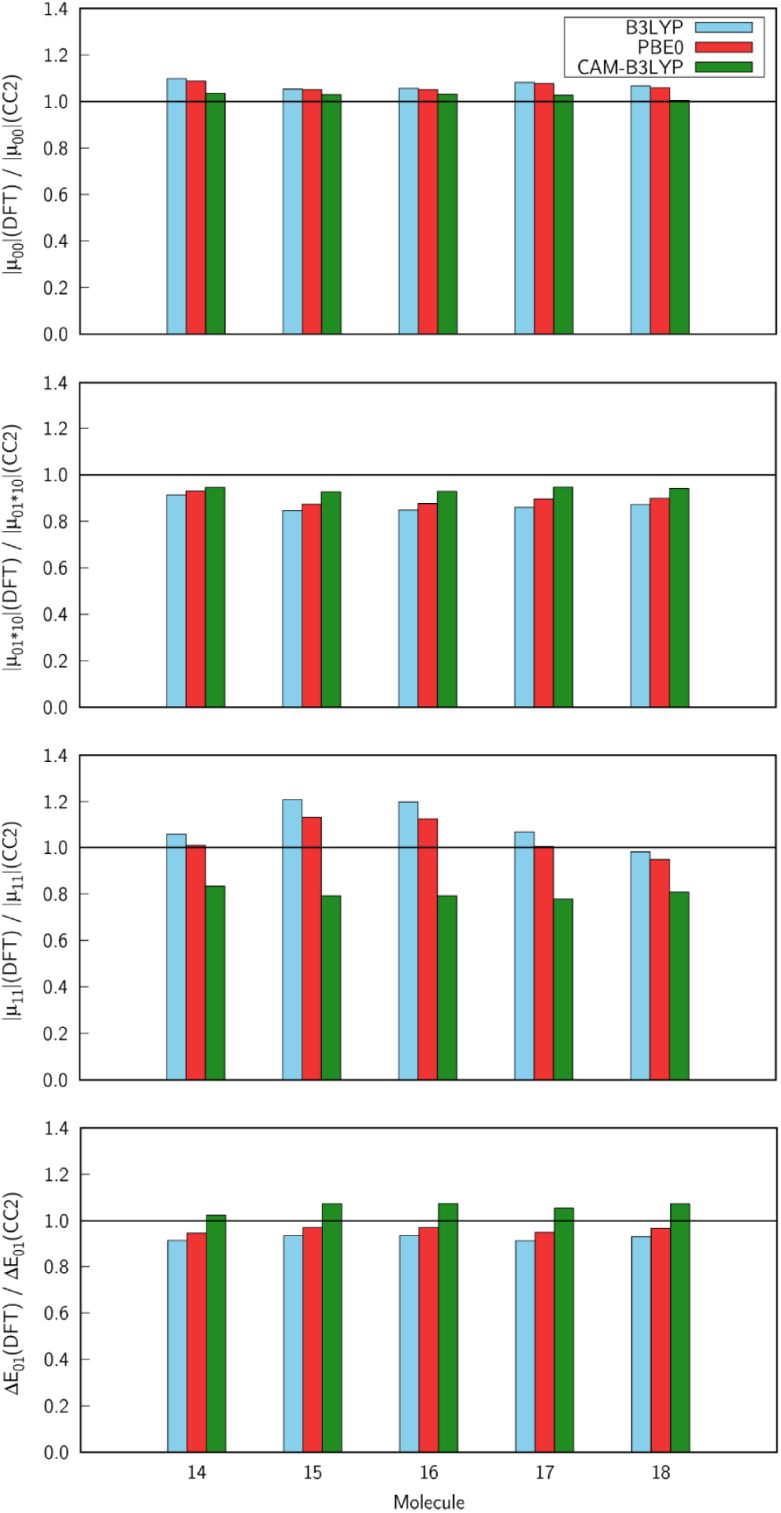
Ratio of the ground state dipole moment (|μ_00_|), right and left transition dipole moment multiplication $\left(|\mu_{01\cdot 10}|=\sqrt{\mu_{x}^{01}\mu_{x}^{10}+\mu_{y}^{01}\mu_{y}^{10}+\mu_{z}^{01}\mu_{z}^{10}}\right)$ , excited state dipole moment (|μ_11_|) and excitation energy (Δ*E*_01_) computed using DFT and CC2 method in connection with the aug-cc-pVDZ basis set.

### Outline

In summary, the performance of B3LYP, PBE0, and CAM-B3LYP functionals in the description of two-photon transition strengths of 18 dye molecules containing difluoroborate moieties against the CC2 method was evaluated. The following conclusions can be drawn from the conducted studies:

- All three exchange-correlation functionals provide δ^2PA^ values that differ significantly from the reference ones, especially in the case of molecules exhibiting the highest 2PA strength, i.e., **14**–**18**. The two-photon transition strengths calculated using CAM-B3LYP fall furthest from the CC2 results and are substantially underestimated. The best agreement with the CC2 method is observed for B3LYP.
- Among all analyzed few-state models, the two-state model, which includes only the ground state 0 and the final excited state 1, provides results closest on average to those predicted by response theory for both CC2 and DFT. Moreover, the values of the two-photon transition strengths calculated within the 2SM using CC2 and CAM-B3LYP methods are similar to those obtained from response theory, in contrast to the results from B3LYP and PBE0 computations.
- The analysis of terms contributing to the two-photon transition strength within the two-state approximation shows that for the vast majority of studied compounds δ_01_ + δ_10_ and δ_11_ contribute the most to δ^2PA^(2SM). Therefore, a proper description of these two terms is important for a correct determination of δ^2PA^(2SM) and, in the case of CAM-B3LYP, which provides reliable values of δ^2PA^(2SM), also for δ^2PA^(RSP).
- CAM-B3LYP underestimates δ_00_, δ_01_, and δ_11_ values of molecules exhibiting the highest 2PA strength, i.e., **13**–**18**. On the contrary, B3LYP and PBE0 do not show such regularity and although they provide results closer to the reference, CAM-B3LYP is the better choice for the description of δ^2PA^(2SM) and δ^2PA^(RSP), as its use allows one to avoid error cancellation.
- The analysis of the spectroscopic parameters for molecules **14**–**18** indicates that the underestimated values of the excited-state dipole moment and overestimated excitation energies are potentially the main sources of the underestimation of δ_01_ and δ_11_ and hence also δ^2PA^(2SM) and δ^2PA^(RSP) by CAM-B3LYP.

The findings of the present work highlight the need for further DFT benchmark studies of two-photon absorption and the great importance of few-state models for the analysis of the behavior of exchange-correlation functionals in predicting 2PA strengths.

## Supplementary Material





## Notes

Values of all computed properties; figures presenting the comparison of results obtained with generalized few-state models and response theory (PDF)
Optimized geometries of all studied molecules (ZIP)

## References

[1] Boyd, R. W. Nonlinear Optics; Academic Press Inc., 1992.

[2] Göppert-Mayer M. (1931). Über
Elementarakte mit zwei Quantensprungen. Ann.
Phys..

[3] Maiman T. H. (1960). Stimulated
Optical Radiation in Ruby. Nature.

[4] He G. S., Tan L.-S., Zheng Q., Prasad P. N. (2008). Multiphoton Absorbing
Materials: Molecular Designs, Characterizations, and Applications. Chem. Rev..

[5] Zareba J. K., Nyk M., Samoć M. (2021). Nonlinear Optical Properties of Emerging Nano- and
Microcrystalline Materials. Adv. Opt. Mater..

[6] Drobizhev M., Makarov N. S., Tillo S. E., Hughes T. E., Rebane A. (2011). Two-photon
absorption properties of fluorescent proteins. Nat. Methods.

[7] Streets A.
M., Li A., Chen T., Huang Y. (2014). Imaging without Fluorescence: Nonlinear
Optical Microscopy for Quantitative Cellular Imaging. Anal. Chem..

[8] Wang B.-G., König K., Halbhuber K.-J. (2010). Two-photon microscopy of deep intravital
tissues and its merits in clinical research. J. Microsc..

[9] Denk W., Strickler J., Webb W. (1990). Two-photon laser scanning fluorescence
microscopy. Science.

[10] Kim H. M., Cho B. R. (2015). Small-Molecule Two-photon
Probes for Bioimaging Applications. Chem. Rev..

[11] Gao D., Agayan R. R., Xu H., Philbert M. A., Kopelman R. (2006). Nanoparticles
for Two-photon Photodynamic Therapy in Living Cells. Nano Lett..

[12] Kim S., Ohulchanskyy Y., Pudavar H. E., Pandey R. K., Prasad P. N. (2007). Organically
Modified Silica Nanoparticles Co-encapsulating Photosensitizing Drug
and Aggregation-enhanced Two-photon Absorbing Fluorescent Dye Aggregates
for Two-photon Photodynamic Therapy. J. Am.
Chem. Soc..

[13] Shen Y., Shuhendler A. J., Ye D., Xu J.-J., Chen H.-Y. (2016). Two-photon
Excitation Nanoparticles for Photodynamic Therapy. Chem. Soc. Rev..

[14] Belfield K. D., Schafer K. J., Liu Y. U., Liu J., Ren X. B., Van Stryland E. W. (2000). Multiphoton-absorbing Organic Materials for Microfabrication,
Emerging Optical Applications and Non-destructive Three-dimensional
Imaging. J. Phys. Org. Chem..

[15] Parthenopoulos D. A., Rentzepis P. M. (1989). Three-Dimensional
Optical Storage Memory. Science.

[16] Dvornikov A. S., Walker E. P., Rentzepis P. M. (2009). Two-Photon
Three-Dimensional Optical
Storage Memory. J. Phys. Chem. A.

[17] Pascal S., David S., Andraud C., Maury O. (2021). Near-infrared dyes
for two-photon absorption in the short-wavelength infrared: Strategies
towards optical power limiting. Chem. Soc. Rev..

[18] Pawlicki M., Collins H. A., Denning R. G., Anderson H. L. (2009). Two-Photon Absorption
and the Design of Two-Photon Dyes. Angew. Chem.,
Int. Ed..

[19] Medishetty R., Zareba J. K., Mayer D., Samoc M., Fischer R. A. (2017). Nonlinear
Optical Properties, Upconversion and Lasing in Metal-organic Frameworks. Chem. Soc. Rev..

[20] Zhou F., Ran X., Fan D., Lu S., Ji W. (2021). Perovskites: Multiphoton
Absorption and Applications. Adv. Opt. Mater..

[21] Ahmed S., Jiang X., Wang C., Kalsoom U., Wang B., Khan J., Muhammad Y., Duan Y., Zhu H., Ren X. (2021). An Insightful Picture of Nonlinear Photonics in 2D
Materials and their Applications: Recent Advances and Future Prospects. Adv. Opt. Mater..

[22] Wielgus M., Zaleśny R., Murugan N. A., Kongsted J., Ågren H., Samoc M., Bartkowiak W. (2013). Two-Photon Solvatochromism II: Experimental
and Theoretical Study of Solvent Effects on the Two-Photon Absorption
Spectrum of Reichardt’s Dye. ChemPhysChem.

[23] Meyers F., Marder S. R., Pierce B. M., Bredas J. L. (1994). Electric-field
Modulated
Nonlinear-optical Properties of Donor-acceptor Polyenes - Sum-over-
states Investigation of the Relationship Between Molecular Polarizabilities
(Alpha, Beta, and Gamma) and Bond-length Alternation. J. Am. Chem. Soc..

[24] Bartkowiak W., Zaleśny R., Leszczynski J. (2003). Relation between bond-length alternation
and two-photon absorption of push-pull conjugated molecules: A quantum-chemical
study. Chem. Phys..

[25] Dreger Z. A., Yang G., White J. O., Li Y., Drickamer H. G. (1998). One- and
Two-photon-pumped Fluorescence from Rhodamine B in Solid Poly­(acrylic
acid) under High Pressure. J. Phys. Chem. B.

[26] Suzuki Y., Tenma Y., Nishioka Y., Kawamata J. (2012). Efficient Nonlinear
Optical Properties of Dyes Confined in Interlayer Nanospaces of Clay
Minerals. Chem. - Asian J..

[27] Kozłowska J., Chołuj M., Zaleśny R., Bartkowiak W. (2017). Two-photon
Absorption of the Spatially Confined LiH Molecule. Phys. Chem. Chem. Phys..

[28] Chakrabarti S., Ruud K. (2009). Molecular Tweezer as a New Promising Class of Compounds for Nonlinear
Optics. Phys. Chem. Chem. Phys..

[29] Beerepoot M. T. P., Alam M. M., Bednarska J., Bartkowiak W., Ruud K., Zaleśny R. (2018). Benchmarking
the performance of exchange-correlation
functionals for predicting two-photon absorption strengths. J. Chem. Theory Comput..

[30] Grabarek D., Andruniów T. (2019). Assessment of Functionals for TDDFT
Calculations of
One- and Two-Photon Absorption Properties of Neutral and Anionic Fluorescent
Proteins Chromophores. J. Chem. Theory Comput..

[31] Chołuj M., Alam M. M., Beerepoot M. T. P., Sitkiewicz S. P., Matito E., Ruud K., Zaleśny R. (2022). Choosing bad
versus worse: Predictions of two-photon-absorption strengths based
on popular density functional approximations. J. Chem. Theory Comput..

[32] Sirimatayananta S., Andruniów T. (2023). Benchmarking two-photon absorption
strengths of rhodopsin
chromophore models with CC3 and CCSD methodologies: An assessment
of popular density functional approximations. J. Chem. Phys..

[33] Ahmadzadeh K., Li X., Rinkevicius Z., Norman P., Zaleśny R. (2024). Toward Accurate
Two-Photon Absorption Spectrum Simulations: Exploring the Landscape
beyond the Generalized Gradient Approximation. J. Phys. Chem. Lett..

[34] Bramantya B., Ośmiałowski B., Zaleśny R. (2025). Computational
insights into two-photon absorption of centrosymmetric organoboron
complexes. Chem. Phys. Lett..

[35] Beerepoot M. T. P., Friese D. H., List N. H., Kongsted J., Ruud K. (2015). Benchmarking
two-photon absorption cross sections: Performance of CC2 and CAM-B3LYP. Phys. Chem. Chem. Phys..

[36] Naim C., Zaleśny R., Jacquemin D. (2024). Two-Photon Absorption Strengths of
Small Molecules: Reference CC3 Values and Benchmarks. J. Chem. Theory Comput..

[37] Ośmiałowski B., Petrusevich E. F., Antoniak M. A., Grela I., Bin Jassar M. A., Nyk M., Luis J. M., Jedrzejewska B., Zaleśny R., Jacquemin D. (2020). Controlling Two-Photon Action Cross Section by Changing
a Single Heteroatom Position in Fluorescent Dyes. J. Phys. Chem. Lett..

[38] Ośmiałowski B., Petrusevich E. F., Nawrot K. C., Paszkiewicz B. K., Nyk M., Zielak J., Jedrzejewska B., Luis J. M., Jacquemin D., Zaleśny R. (2021). Tailoring the nonlinear absorption of fluorescent dyes
by substitution at a boron center. J. Mater.
Chem. C.

[39] Frisch, M. J. ; Trucks, G. W. ; Schlegel, H. B. ; Scuseria, G. E. ; Robb, M. A. ; Cheeseman, J. R. ; Scalmani, G. ; Barone, V. ; Petersson, G. A. ; Nakatsuji, H. ; Gaussian 16, Revision B.01; Gaussian Inc., 2016.

[40] Lee C., Yang W., Parr R. G. (1988). Development
of the Colle-Salvetti
correlation-energy formula into a functional of the electron density. Phys. Rev. B.

[41] Becke A. D. (1993). Density-functional
thermochemistry. III. The role of exact exchange. J. Chem. Phys..

[42] Stephens P. J., Devlin F. J., Chabalowski C. F., Frisch M. J. (1994). Ab Initio Calculation
of Vibrational Absorption and Circular Dichroism Spectra Using Density
Functional Force Fields. J. Phys. Chem..

[43] Adamo C., Barone V. (1999). Toward reliable density functional methods without
adjustable parameters: The PBE0 model. J. Chem.
Phys..

[44] Yanai T., Tew D. P., Handy N. C. (2004). A new hybrid
exchange-correlation
functional using the Coulomb-attenuating method (CAM-B3LYP). Chem. Phys. Lett..

[45] Dunning T. H. (1989). Gaussian
basis sets for use in correlated molecular calculations. I. The atoms
boron through neon and hydrogen. J. Chem. Phys..

[46] Schmidt M. W., Baldridge K. K., Boatz J. A., Elbert S. T., Gordon M. S., Jensen J. H., Koseki S., Matsunaga N., Nguyen K. A., Su S. (1993). General atomic and molecular
electronic structure system. J. Comput. Chem..

[47] Jacquemin D., Wathelet V., Perpéte E. A., Adamo C. (2009). Extensive TD-DFT Benchmark:
Singlet-Excited States of Organic Molecules. J. Chem. Theory Comput..

[48] Laurent A., Jacquemin D. (2013). TD-DFT benchmarks: A review. Int. J. Quantum Chem..

[49] Le
Guennic B., Jacquemin D. (2015). Taking Up the Cyanine Challenge with
Quantum Tools. Acc. Chem. Res..

[50] Charaf-Eddin A., Planchat A., Mennucci B., Adamo C., Jacquemin D. (2013). Choosing a
Functional for Computing Absorption and Fluorescence Band Shapes with
TD-DFT. J. Chem. Theory Comput..

[51] Peach M. J. G., Benfield P., Helgaker T., Tozer D. J. (2008). Excitation energies
in density functional theory: An evaluation and a diagnostic test. J. Chem. Phys..

[52] Friese D. H., Hättig C., Ruud K. (2012). Calculation of two-photon absorption
strengths with the approximate coupled cluster singles and doubles
model CC2 using the resolution-of-identity approximation. Phys. Chem. Chem. Phys..

[53] TURBOMOLE V7.0 2015, a development of University of Karlsruhe and Forschung-szentrum Karlsruhe GmbH, 1989–2007, TURBOMOLE GmbH, http://www.turbomole.com 2007.

[54] Weigend F., Köhn A., Hättig C. (2002). Efficient use of the correlation
consistent basis sets in resolution of the identity MP2 calculations. J. Chem. Phys..

