# Supplementary material for: Performance of Density Functional Approximations in Calculations of Electronic Two-Photon Transition Strengths of Fluorescent Dyes
Source: J Phys Chem A. 2025 May 24;129(22):4903–10. doi: 10.1021/acs.jpca.5c01509 (PMC12147207; doi:10.1021/acs.jpca.5c01509)
Supplement: Supplementary file 1 [file jp5c01509_si_001.pdf]

Supporting information

Performance of Density Functional Approximations in Calculations of Electronic Two-Photon Transition Strengths of Fluorescent Dyes

Marta Chołuj

Department of Physical and Quantum Chemistry, Faculty of Chemistry, Wrocław University of Science and Technology, Wybrzeże Wyspiańskiego 27, Wrocław 50-370, Poland

E-mail: marta.choluj@pwr.edu.pl

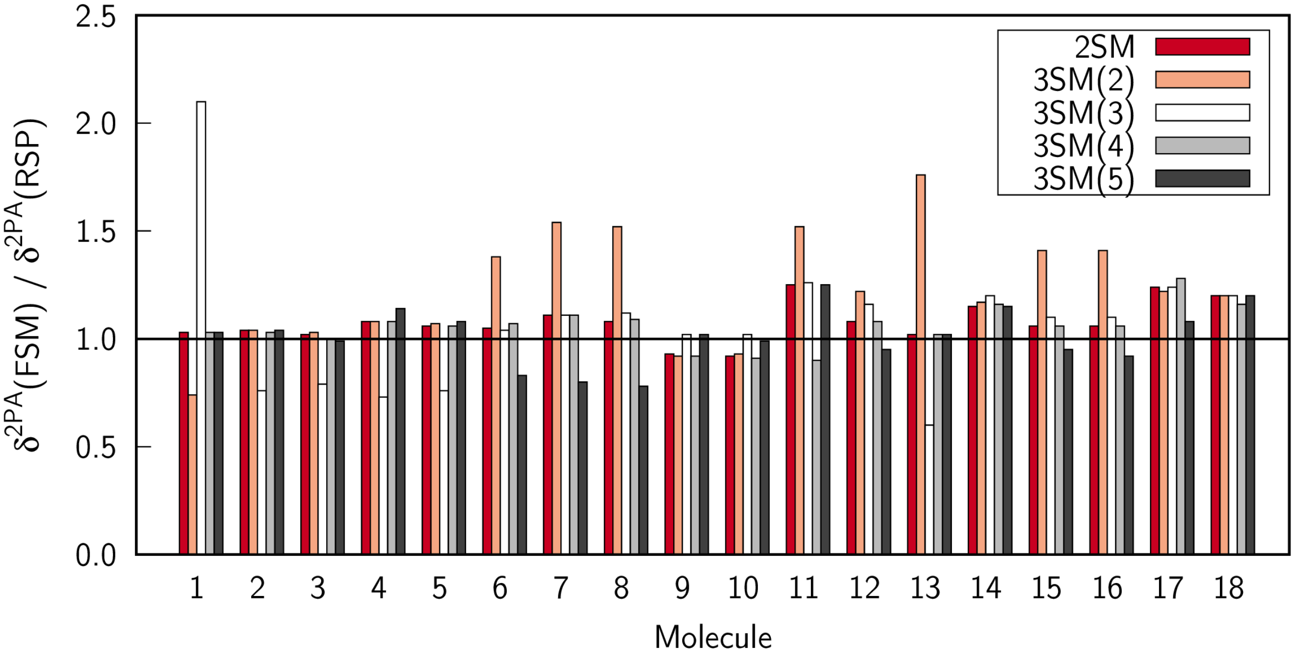

Figure S1 Ratios between the two-photon transition strengths δ^{2PA} computed with the few-state models (FSM), i.e. a two-state model (2SM) as well as a three-state model with additional state 2, 3, 4 or 5 (3SM(2), 3SM(3), 3SM(4), 3SM(5), respectively) and with response theory (RSP) at CC2/aug-cc-pVDZ level of theory.

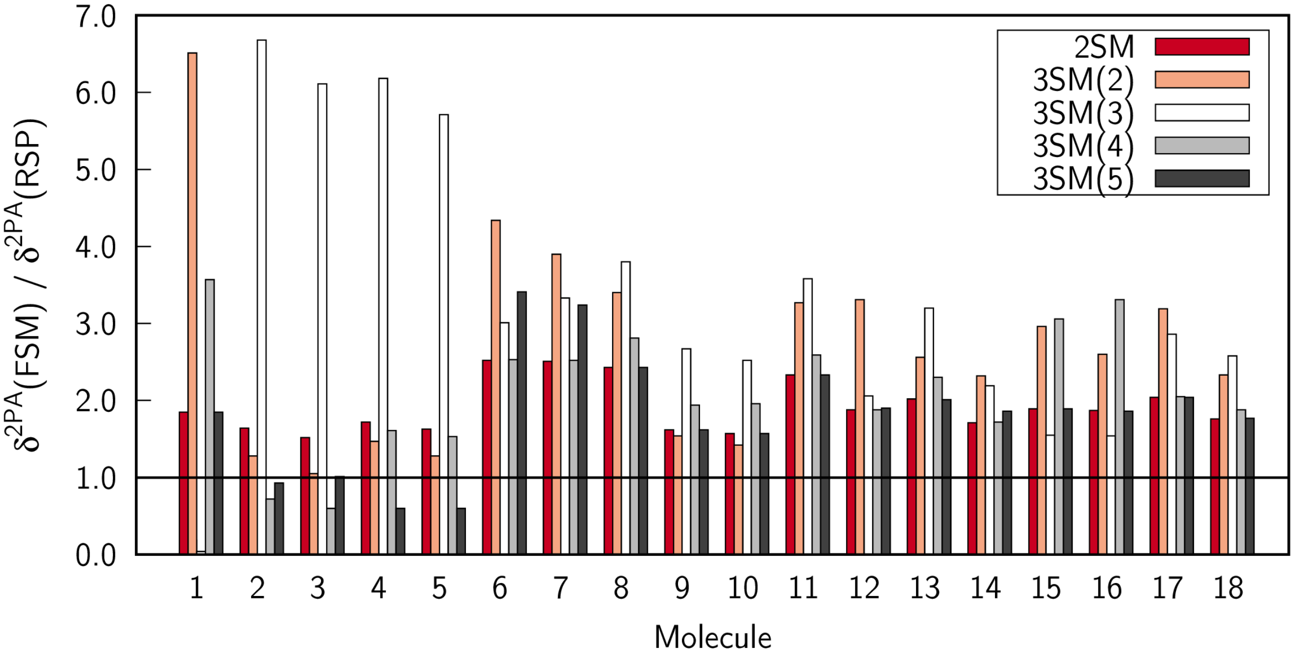

Figure S2 Ratios between the two-photon transition strengths δ^{2PA} computed with the few-state models (FSM), i.e. a two-state model (2SM) as well as a three-state model with additional state 2, 3, 4 or 5 (3SM(2), 3SM(3), 3SM(4), 3SM(5), respectively) and with response theory (RSP) at B3LYP/aug-cc-pVDZ level of theory.

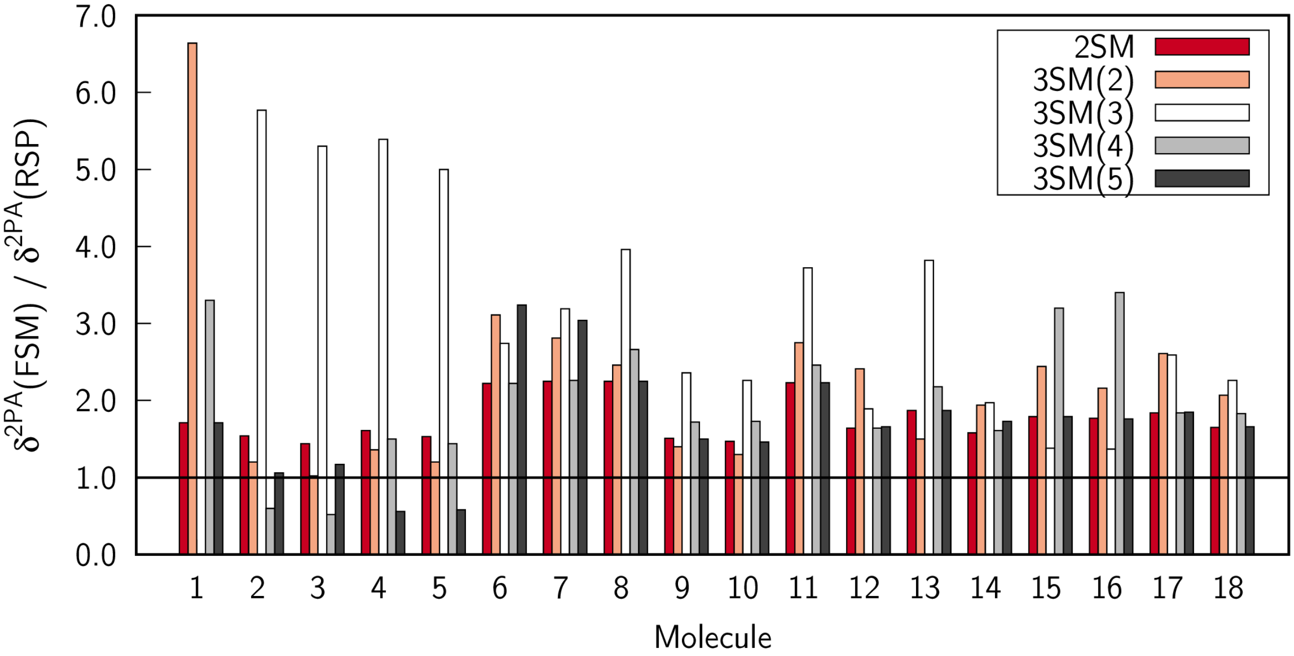

Figure S3 Ratios between the two-photon transition strengths δ^{2PA} computed with the few-state models (FSM), i.e. a two-state model (2SM) as well as a three-state model with additional state 2, 3, 4 or 5 (3SM(2), 3SM(3), 3SM(4), 3SM(5), respectively) and with response theory (RSP) at PBE0/aug-cc-pVDZ level of theory.

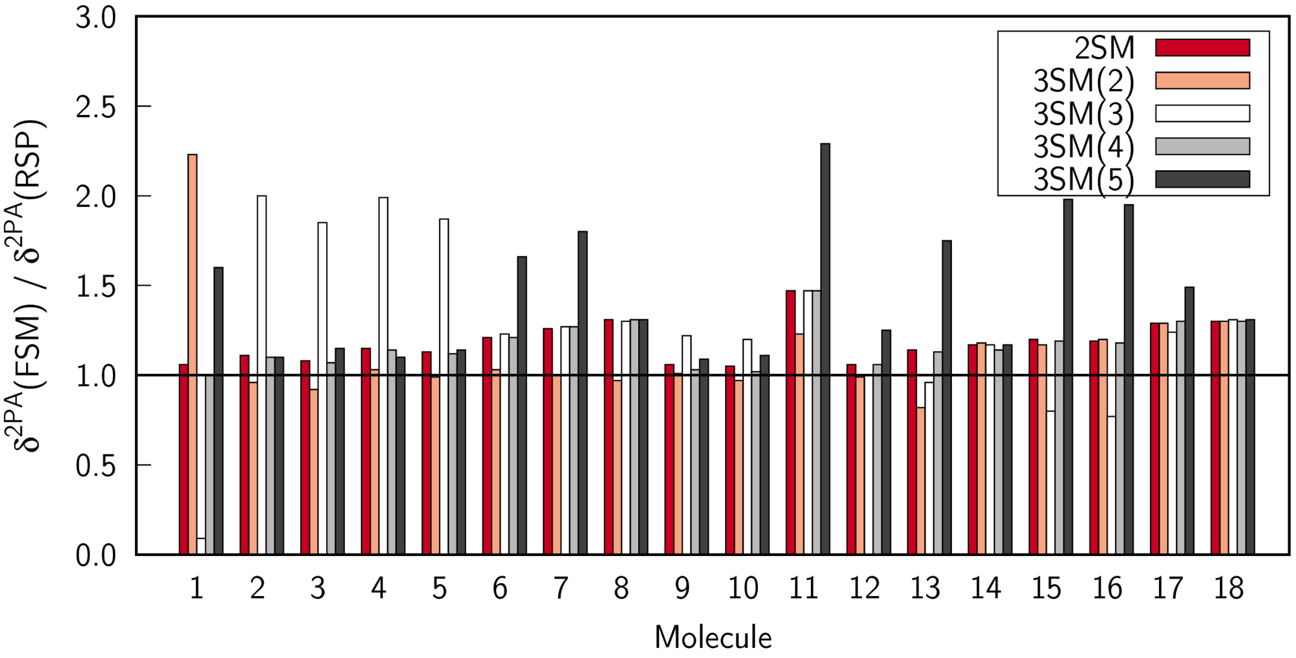

Figure S4 Ratios between the two-photon transition strengths δ^{2PA} computed with the few-state models (FSM), i.e. a two-state model (2SM) as well as a three-state model with additional state 2, 3, 4 or 5 (3SM(2), 3SM(3), 3SM(4), 3SM(5), respectively) and with response theory (RSP) at CAM-B3LYP/aug-cc-pVDZ level of theory.

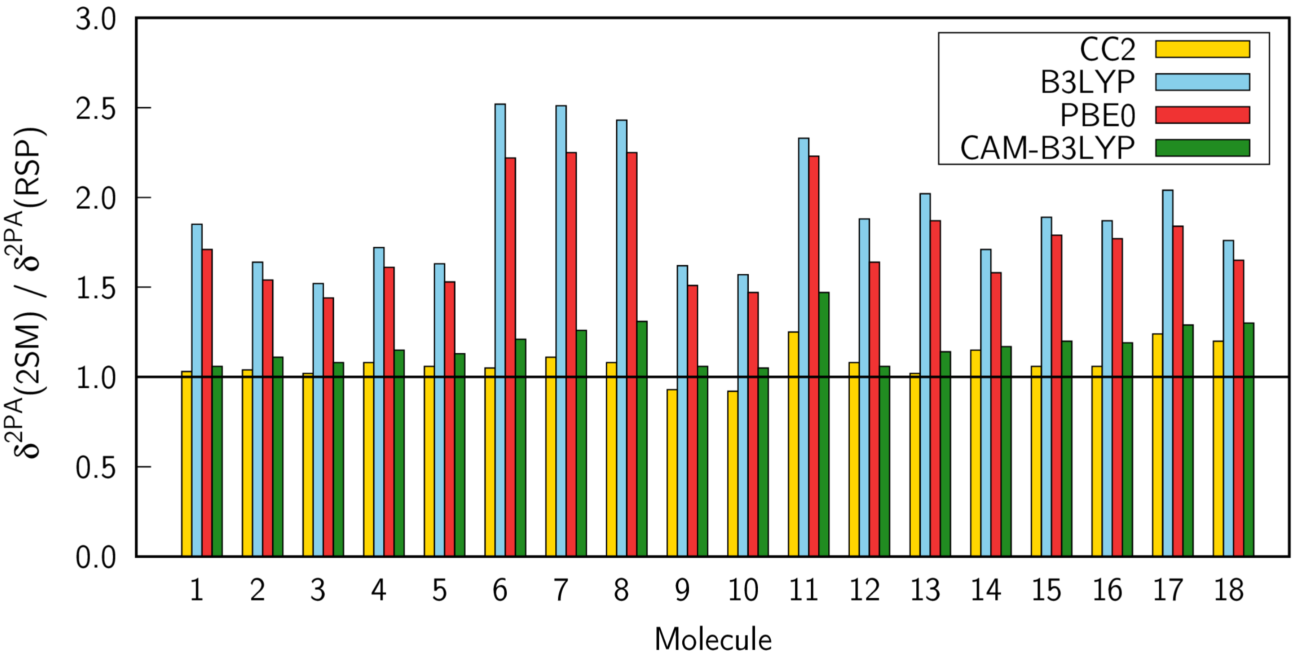

Figure S5 Ratios between the two-photon transition strengths δ^{2PA} computed with a two-state model (2SM) and with response theory (RSP) using aug-cc-pVDZ basis set.

Table S1 Two-photon transition strengths (δ^{2PA}, in a.u.) computed with response theory using aug-cc-pVDZ basis set.

	δ^{2PA}				$\delta^{2PA}(DFT)/\delta^{2PA}(CC2)$		
Molecule	CC2	B3LYP	PBE0	CAM-B3LYP	B3LYP	PBE0	CAM-B3LYP
1	2558	2500	2074	1192	0.977	0.811	0.466
2	2624	1583	1402	1001	0.603	0.534	0.381
3	2758	1687	1496	1064	0.612	0.542	0.386
4	2998	2170	1888	1309	0.724	0.630	0.437
5	3174	2315	2017	1393	0.729	0.635	0.439
6	3981	4534	3807	1885	1.139	0.956	0.473
7	6478	6776	5753	2729	1.046	0.888	0.421
8	6955	12816	10660	4033	1.843	1.533	0.580
9	7474	6576	5537	2918	0.880	0.741	0.390
10	7820	6853	5779	3060	0.876	0.739	0.391
11	14573	20027	17579	7238	1.374	1.206	0.497
12	16753	14040	12024	6967	0.838	0.718	0.416
13	20778	19660	16429	6427	0.946	0.791	0.309
14	26885	19557	17363	10614	0.727	0.646	0.395
15	36952	28362	24660	10765	0.768	0.667	0.291
16	38131	29112	25320	11126	0.763	0.664	0.292
17	67134	41107	37324	21341	0.612	0.556	0.318
18	86142	44021	40906	26576	0.511	0.475	0.309

Table S2 The procentage contributions of δ_{00}, $\delta_{01+10} = \delta_{01} + \delta_{10}$ and δ_{11} terms to the two-photon transition strength within a two-state approximation.

	CC2			B3LYP			PBE0			CAM-B3LYP		
Molecule	δ_{00}	δ_{01+10}	δ_{11}	δ_{00}	δ_{01+10}	δ_{11}	δ_{00}	δ_{01+10}	δ_{11}	δ_{00}	δ_{01+10}	δ_{11}
1	224	-350	226	122	-212	189	156	-272	216	437	-778	441
2	138	-93	55	128	-92	64	150	-122	72	316	-365	149
3	127	-82	55	123	-90	66	143	-116	73	291	-333	142
4	65	-88	122	58	-105	147	70	-127	158	142	-250	209
5	62	-88	126	58	-112	154	69	-133	164	137	-251	214
6	19	4	77	5	2	92	7	0	93	31	-19	88
7	11	-14	103	4	-11	107	6	-14	109	21	-36	115
8	27	21	52	5	16	79	6	18	76	33	8	59
9	57	-128	171	39	-104	165	48	-125	177	124	-270	246
10	55	-130	175	40	-110	170	49	-131	182	122	-274	252
11	6	14	79	2	3	95	2	4	94	10	8	83
12	17	-84	167	17	-91	175	22	-109	187	44	-174	230
13	38	-128	191	20	-89	169	26	-106	180	96	-281	285
14	43	-210	267	49	-231	282	57	-258	300	98	-384	386
15	26	-132	205	18	-103	185	22	-117	195	60	-233	273
16	27	-134	207	19	-107	188	23	-121	198	61	-238	277
17	22	-127	204	25	-135	211	30	-154	225	57	-242	285
18	51	-240	289	69	-297	328	77	-321	344	117	-440	424

Table S3 The δ_{00} term (in a.u.) contributing to the two-photon transition strength within a two-state approximation.

	δ_{00}				δ_{00}(DFT)/δ_{00}(CC2)		
Molecule	CC2	B3LYP	PBE0	CAM-B3LYP	B3LYP	PBE0	CAM-B3LYP
1	5887	5664	5529	5503	0.962	0.939	0.935
2	3757	3334	3233	3502	0.887	0.860	0.932
3	3549	3173	3079	3353	0.894	0.868	0.945
4	2115	2166	2107	2132	1.024	0.996	1.008
5	2073	2192	2123	2146	1.057	1.024	1.035
6	794	623	633	709	0.785	0.797	0.893
7	780	694	712	714	0.891	0.913	0.915
8	2003	1484	1543	1748	0.741	0.770	0.873
9	3974	4162	4037	3837	1.047	1.016	0.965
10	4006	4289	4139	3914	1.071	1.033	0.977
11	1186	826	847	1036	0.696	0.714	0.873
12	3066	4469	4307	3251	1.458	1.405	1.060
13	7991	8121	8022	7099	1.016	1.004	0.888
14	13279	16549	15723	12244	1.246	1.184	0.922
15	10385	9813	9703	7781	0.945	0.934	0.749
16	10803	10318	10146	8142	0.955	0.939	0.754
17	18760	20677	20508	15676	1.102	1.093	0.836
18	52920	53374	51592	40429	1.009	0.975	0.764

Table S4 The δ_{01} term (in a.u.) contributing to the two-photon transition strength within a two-state approximation.

	δ_{01}				$\delta_{01}(\text{DFT})/\delta_{01}(\text{CC2})$		
Molecule	CC2	B3LYP	PBE0	CAM-B3LYP	B3LYP	PBE0	CAM-B3LYP
1	-4589	-4895	-4820	-4898	1.067	1.050	1.067
2	-1265	-1196	-1314	-2023	0.945	1.039	1.599
3	-1143	-1151	-1250	-1916	1.007	1.093	1.676
4	-1413	-1962	-1927	-1883	1.388	1.363	1.332
5	-1479	-2106	-2043	-1968	1.424	1.381	1.330
6	79	120	-4	-214	1.521	-0.049	-2.701
7	-498	-952	-933	-617	1.912	1.873	1.239
8	792	2489	2099	208	3.143	2.651	0.263
9	-4458	-5555	-5227	-4182	1.246	1.173	0.938
10	-4706	-5947	-5551	-4396	1.264	1.179	0.934
11	1301	815	815	402	0.627	0.626	0.309
12	-7577	-12088	-10772	-6430	1.595	1.422	0.849
13	-13645	-17639	-16326	-10340	1.293	1.197	0.758
14	-32478	-38779	-35344	-23920	1.194	1.088	0.736
15	-25917	-27781	-25908	-15067	1.072	1.000	0.581
16	-27055	-29024	-26981	-15791	1.073	0.997	0.584
17	-52759	-56731	-53126	-33379	1.075	1.007	0.633
18	-124087	-114773	-108003	-76254	0.925	0.870	0.615

Table S5 The δ_{11} term (in a.u.) contributing to the two-photon transition strength within a two-state approximation.

	δ_{11}				δ_{11}(DFT)/δ_{11}(CC2)		
Molecule	CC2	B3LYP	PBE0	CAM-B3LYP	B3LYP	PBE0	CAM-B3LYP
1	5915	8750	7655	5551	1.479	1.294	0.938
2	1490	1656	1550	1655	1.111	1.040	1.110
3	1538	1702	1580	1631	1.107	1.028	1.061
4	3941	5494	4778	3138	1.394	1.212	0.796
5	4247	5786	5046	3358	1.363	1.188	0.791
6	3210	10567	7840	1997	3.291	2.442	0.622
7	7400	18242	14093	3955	2.465	1.905	0.534
8	3941	24696	18246	3128	6.266	4.629	0.794
9	11904	17620	14756	7627	1.480	1.240	0.641
10	12634	18395	15431	8087	1.456	1.221	0.640
11	14485	44198	36739	8818	3.051	2.536	0.609
12	30136	46155	36937	16996	1.532	1.226	0.564
13	40575	66786	55404	20937	1.646	1.365	0.516
14	82659	94547	82395	48055	1.144	0.997	0.581
15	80761	99464	86240	35272	1.232	1.068	0.437
16	83747	102074	88522	36709	1.219	1.057	0.438
17	170166	176581	154601	78679	1.038	0.909	0.462
18	298548	253447	231726	146704	0.849	0.776	0.491

Table S6 The ground state dipole moment ($|\mu_{00}|$), right and left transition moment multiplication ($|\mu_{01*10}| = \sqrt{\mu_x^{01}\mu_x^{10} + \mu_y^{01}\mu_y^{10} + \mu_z^{01}\mu_z^{10}}$), excited state dipole moment ($|\mu_{11}|$) and excitation energy (ΔE_{01}), in a.u., computed using DFT and CC2 method in connection with the aug-cc-pVDZ basis set.

Molecule	CC2	B3LYP	PBE0	CAM-B3LYP	B3LYP	PBE0	CAM-B3LYP
			$\|\mu_{00}\|$			$\|\mu_{00}\|$(DFT)/$\|\mu_{00}\|$(CC2)	
14	2.4929	2.7370	2.7134	2.5839	1.098	1.088	1.036
15	2.2209	2.3377	2.3333	2.2854	1.053	1.051	1.029
16	2.2520	2.3772	2.3680	2.3227	1.056	1.051	1.031
17	2.7576	2.9839	2.9704	2.8309	1.082	1.077	1.027
18	3.8111	4.0646	4.0341	3.8290	1.067	1.059	1.005
			$\|\mu_{01*10}\|$			$\|\mu_{01*10}\|$(DFT)/$\|\mu_{01*10}\|$(CC2)	
14	3.8760	3.5382	3.6042	3.6680	0.913	0.930	0.946
15	3.8431	3.2545	3.3605	3.5642	0.847	0.874	0.927
16	3.8242	3.2482	3.3516	3.5522	0.849	0.876	0.929
17	4.2089	3.6191	3.7726	3.9879	0.860	0.896	0.947
18	4.2702	3.7252	3.8341	4.0171	0.872	0.898	0.941
			$\|\mu_{11}\|$			$\|\mu_{11}\|$(DFT)/$\|\mu_{11}\|$(CC2)	
14	5.6845	6.0317	5.7346	4.7350	1.061	1.009	0.833
15	4.8491	5.8557	5.4957	3.8415	1.208	1.133	0.792
16	4.9424	5.9238	5.5636	3.9152	1.199	1.126	0.792
17	6.8805	7.3609	6.9052	5.3454	1.070	1.004	0.777
18	8.5198	8.3570	8.0740	6.8825	0.981	0.948	0.808
			ΔE_{01}			ΔE_{01}(DFT)/ΔE_{01}(CC2)	
14	0.13284	0.12172	0.12584	0.13599	0.916	0.947	1.024
15	0.11272	0.10566	0.10950	0.12095	0.937	0.971	1.073
16	0.11249	0.10538	0.10923	0.12080	0.937	0.971	1.074
17	0.12109	0.11081	0.11516	0.12774	0.915	0.951	1.055
18	0.11697	0.10907	0.11320	0.12552	0.932	0.968	1.073
